# Comparison of Newly Diagnosed and Relapsed Patients with Acute Promyelocytic Leukemia Treated with Arsenic Trioxide: Insight into Mechanisms of Resistance

**DOI:** 10.1371/journal.pone.0121912

**Published:** 2015-03-30

**Authors:** Ezhilarasi Chendamarai, Saravanan Ganesan, Ansu Abu Alex, Vandana Kamath, Sukesh C. Nair, Arun Jose Nellickal, Nancy Beryl Janet, Vivi Srivastava, Kavitha M. Lakshmi, Auro Viswabandya, Aby Abraham, Mohammed Aiyaz, Nandita Mullapudi, Raja Mugasimangalam, Rose Ann Padua, Christine Chomienne, Mammen Chandy, Alok Srivastava, Biju George, Poonkuzhali Balasubramanian, Vikram Mathews

**Affiliations:** 1 Department of Haematology, Christian Medical College, Vellore, India; 2 Department of Transfusion Medicine and Immunohaematology, Christian Medical College, Vellore, India; 3 Department of Biochemistry, Christian Medical College, Vellore, India; 4 Cytogenetics Unit, Christian Medical College, Vellore, India; 5 Genotypic Technology, Bengaluru, India; 6 UMR 1131 Institut d’Hématologie, Hôpital Saint Louis, I avenue Claude Vellefaux, 75010 Paris, France; University of Heidelberg, GERMANY

## Abstract

There is limited data on the clinical, cellular and molecular changes in relapsed acute promyeloytic leukemia (RAPL) in comparison with newly diagnosed cases (NAPL). We undertook a prospective study to compare NAPL and RAPL patients treated with arsenic trioxide (ATO) based regimens. 98 NAPL and 28 RAPL were enrolled in this study. RAPL patients had a significantly lower WBC count and higher platelet count at diagnosis. IC bleeds was significantly lower in RAPL cases (P=0.022). The ability of malignant promyelocytes to concentrate ATO intracellularly and their *in-vitro* IC50 to ATO was not significantly different between the two groups. Targeted NGS revealed PML B2 domain mutations in 4 (15.38%) of the RAPL subset and none were associated with secondary resistance to ATO. A microarray GEP revealed 1744 genes were 2 fold and above differentially expressed between the two groups. The most prominent differentially regulated pathways were cell adhesion (n=92), cell survival (n=50), immune regulation (n=74) and stem cell regulation (n=51). Consistent with the GEP data, immunophenotyping revealed significantly increased CD34 expression (P=0.001) in RAPL cases and there was in-vitro evidence of significant microenvironment mediated innate resistance (EM-DR) to ATO. Resistance and relapse following treatment with ATO is probably multi-factorial, mutations in PML B2 domain while seen only in RAPL may not be the major clinically relevant cause of subsequent relapses. In RAPL additional factors such as expansion of the leukemia initiating compartment along with EM-DR may contribute significantly to relapse following treatment with ATO based regimens.

## Introduction

Significant strides have been made in the management of acute promyelocytic leukemia (APL) in the last two decades[1]. There has been an evolution in the treatment of APL towards reduced intensity and low toxicity regimens by the use of targeted therapeutic agents. This was facilitated by the improved understanding of cellular and molecular biology of this condition and recognition of mechanism of action of some of the non chemotherapeutic agents used to treat this condition[2, 3]. Key agents that have contributed to reduced intensity of chemotherapy have been the use of all-trans retinoic acid (ATRA)[4] and arsenic trioxide (ATO) either alone or in combination[5, 6]. While there is significant understanding of the mechanisms of resistance to ATRA[7] there is limited data on the potential mechanisms of resistance to ATO, especially that which is relevant in the clinic.

The treatment of newly diagnosed APL patients (NAPL) with either single agent ATO or ATO combined with ATRA has been reported to be associated with durable remissions and minimal toxicity, with results being comparable to that achieved with conventional chemotherapy used for this disease[8–10]. Despite its efficacy in the treatment of APL it has been noted that 10–20% of newly diagnosed and 30–50% of relapsed patients (RAPL) will relapse, mostly within the high risk subset, after treatment with ATO based regimens[11, 12]. In patients with RAPL, ATO is effective in inducing molecular remissions in the majority without the toxicity profile of combination chemotherapy and it does not have cross resistance with ATRA[9, 12]. However, in the absence of consolidation with an autologous stem cell transplantation (SCT) there is a high incidence of relapse[13]. There is limited data to explain this inferior long term clinical response in RAPL treated with ATO based regimens in contrast to NAPL.

As with other malignancies, it is likely that patients with RAPL have acquired additional cytogenetic, molecular and cellular defects that accounts for these differences which have not been well characterized[14–16]. Potential mechanisms of relapse in APL treated with ATO could be related to clonal evolution leading to ATO resistance or resistance in a poorly defined leukemia initiating compartment. Recently it was recognized that ATO directly binds to the B2 domain of the PML-RARA oncoprotein which leads to its SUMOylation and subsequent degradation [17]. More significantly it was reported that mutations in the same domain leads to resistance to ATO [18]. There is limited data on the frequency of these mutations in a cohort of patients with RAPL previously treated with ATO. It is also recognized that majority of RAPL patients do not have any mutations in the PML-RARA gene[19]. The role of microenvironment-mediated drug resistance (EM-DR) in APL treated with ATO is not known.

At our center we have been using a single agent ATO based regimen for NAPL for more than a decade. We were hence in a unique position to compare NAPL treated with such a regimen and compare acquired changes in patients who subsequently relapsed.

## Materials and Methods

### Patients and Samples

From May 2007 to December 2011, bone marrow and peripheral blood samples from NAPL and RAPL (hematological relapses only) admitted at our center were enrolled in this prospective study. Institutional review board approved the study design and the consent forms. Written and informed consent was obtained from all enrolled cases. (Institutional Review Board of Christian Medical College, Vellore, India: IRB, CMC, Vellore. RC Min 5884. 18th April, 2006). NAPL were treated with a single agent ATO based regimen as has been previously reported by us[11, 20]. The clinical outcome in RAPL consolidated with and without an autologous SCT following induction of molecular remission has also been previously reported by us[13]. The human APL cell line NB4[21] (Kind gift from Dr Harry Iland, RPAH, Sydney, Australia with permission from Dr Michel Lanotte) was used for some experiments. HUVEC cell line (ATCC, Manassas, USA) was used as a control for stromal cells in the co-culture experiments. An ATO resistance cell line (NB4EV AsR1) was derived from the NB4 cell line in-house and was also used as a control for some experiments.

### Enrichment of Promyelocytes by Lineage Depletion using VarioMACS

Patient samples having <80% blasts were enriched by negative selection using lineage depletion cocktail in a VarioMACS system (MitenyiBiotec, Gladbach, Germany) and were purified to target ≥90% blasts for experiments, where this was required.

### Morphology, immunophenotype (IPT) and cytogenetic (CTG) evaluation

These were done using standard established methods (S1 File Methods A to C).

### Intracellular Arsenic trioxide Concentration

The intracellular level of arsenic was measured by atomic absorption spectrometry in the blasts using well established and published protocols [22, 23]. Details of the method and steps taken to standardize the assay are provided in the S1 File: Methods D.

### In-vitro ATO Cytotoxicity Assay

An in-vitro evaluation of ATO sensitivity was done using an MTT assay system (Biotium, Inc. CA, USA) (S1 File: Methods E).

### Screening for PML mutations using targeted Ion torrent PGM next generation sequencing (NGS)

Targeted customized exome sequencing was done for PML-RARA transcripts using cDNAs from 27 NAPL and 25 RAPL cases. Library preparation and Ion PGM sequencing were performed following certified protocols (Life-technologies, Carlsbad, CA, USA) at Genotypic Technology genomics facility (Bengaluru, India). A two step nested RT-PCR was performed to amplify B2 domain of the PML transcripts using in house generated primers (S1 File: Methods F: Details of library preparation, quality assessment steps are also given). The reads were aligned to the reference PML amplicon using TMAP algorithm and variants were detected by the plugin Variant caller (v) of Torrent Suite v3.6.2.

### Microarray Analysis for Gene Expression Profiling (GEP)

Patients bone marrow samples at diagnosis and at relapse with ≥ 90% blasts or enriched samples were used for microarray studies (Genotypic Technology, Bengaluru). Gene expression microarray was done using 44k human microarray chip analysis (Agilent technologies) (S1 File: Methods G).

### Validation of microarray data

To validate the results obtained by microarray with an independent method we did real-time RT-PCR (RQ-PCR) using commercially available ABI TaqMan assay system (Applied Biosystems, Darmstadt, Germany). We randomly chose 25 genes from the differentially regulated list and assessed their expression levels in the samples from the 8 unmatched newly diagnosed and relapsed cases on (samples on which the microarray analysis was initially done). We also did the RQ-PCR analysis for the same 25 genes in a second validation set of 10 patients for whom sequential samples at diagnosis and relapse were subsequently available. The expression levels of the target genes and an endogenous control gene (GAPDH) were evaluated. The relative expression of these target genes at diagnosis and at relapse was calculated by 2^-ΔΔCT^ method and expressed as fold difference.

### Impact of Stromal Interaction with Malignant Promyelocytes

After getting written informed consent third party mesenchymal stromal cells (MSC) were isolated from an aliquot of bone marrow taken from healthy allogeneic donors. For all experiments MSCs from passages 3 and 4 were used. These experiments were also repeated using a stromal cell line (HS-5). NB4 cell lines or primary APL cells (1x10^5^cells/well) were added on a layer of MSCs (at least 70% confluent at time of experiment). The co-culture system was left in the incubator overnight and then exposed to 2μM, 4μM and 6μM ATO concentrations. Appropriate controls with and without stromal cells were included. After 48 hour incubation at 37°C in a CO_2_ incubator, Annexin V apoptosis assay was performed as per standard and established immune-phenotyping protocols (The data generated was compared to untreated cells maintained for the same period which for standardization was taken as 100%). Further evaluation of impact of co-culture on cell cycle analysis, cell proliferation assays, changes in the surface marker expression and the effect of blocking VLA-4 and VLA-5 integrin on the chemosensitivity was done on NB4 cells. (S1 File: Methods H).

### Statistical Analysis

The χ2 or fisher exact test and Mann Whitney U test were used to compare differences between groups for clinical and laboratory parameters. The probability of survival was estimated with the use of the product-limit method of Kaplan-Meier for overall survival (OS), event free survival (EFS) was compared by the log rank test. All survival estimates are reported as ± 1SE. All P-values were 2-sided, with values of 0.05 or less indicating statistical significance. Statistical analysis used the SPSS 16.0 Software (Chicago, USA). Non linear regression curves were done with GraphPad Prism Software V5 (California, USA).

## Results

### Patient accrual and the baseline demographic data

Ninety eight (78%) were NAPL and 28 (22%) were RAPL. Among the 28 RAPL, twenty three of them were first relapse and 5 were second relapses. Eleven of the 28 relapse cases had matched samples available at diagnosis. All 28 had relapsed in the bone marrow with a median blast percentage of 82% (range: 26–98), while 2 cases had additional extra-medullary relapse in the central nervous system. Table 1 summarizes the clinical comparison between NAPL and RAPL. Briefly, the median age of NAPL and RAPL cases were not significantly different. The number of males in the RAPL group was significantly higher. RAPL had a significantly lower white cell count (P <0.0001) and a significantly higher platelet count (P = 0.006) at the time of diagnosis in comparison to NAPL. There was less coagulopathy in the RAPL in comparison to NAPL as evidenced by the significantly lower number of intra-cranial bleeds (P = 0.022) and the significantly lower number of platelet units utilized during induction therapy (P = 0.027). The possibility of a lead time bias resulting in earlier diagnosis of relapsed patients (on regular follow up) compared to newly diagnosed patients resulting in lower white cell counts and coagulopathy cannot be excluded.

**Table 1:** Comparison of the clinical and demographic data of the newly diagnosed patients Vs. relapsed acute promyelocytic leukemia patients.

Demographic Parameters	Newly Diagnosed N = 98 N (%) / Median (Range)	Relapsed N = 28 N (%) / Median (Range)	P-value
Age (years)	28 (2–60)	31 (8–54)	0.997
Sex: Male	48 (49)	25 (89.3)	0.000
WBC x 10^9^/Lt	14.5 (0.5–290)	3.5 (0.5–24.90)	0.000
Platelet x 10^9^/Lt	17 (3–85)	31.5 (6–248)	0.006
RT-PCR	-	-	-
bcr1	59 (60.2)	21(75)	0.311
bcr2	2 (2)	0	
bcr3	37 (37.8)	7 (25)	
Lactate dehydrogenase (IU/Lt))	748 (291–2700)	554 (284–1155)	0.006
Fibrinogen (mg/dl)	161 (25–689)	163 (70–1122)	0.975
Additional CTG finding	-	-	-
Yes	34 (36.6)	11 (61.1)	0.068
No	59 (63.4)	7 (38.9)	
IC Bleed (at diagnosis or during induction therapy)	-	-	-
Yes	16 (16.3)	0	0.022
No	82 (83.7)	28 (100)	
Differentiation syndrome (during induction therapy)	-	-	-
Yes	13 (13.3)	2 (7.11)	0.518
No	85 (86.7)	26 (92.9)	
Blood product utilization induction	-	-	-
PRC^^^ units	24 (0–85)	16 (0–48)	0.027
FFP* units	12 (0–72)	7 (0–60)	0.156
Cryoprecipitate units	6 (0–59)	3 (0–30)	0.456

^^^ PRC = platelet rich concentrate

*FFP = Fresh frozen plasma

### Comparison of cellular and in-vitro ATO sensitivity

Potential morphological distinguishing features between the NAPL and RAPL samples were reviewed in 20 random blinded samples from the NAPL and RAPL group by two independent hemato-pathologists. The consensus was that there were no distinguishing morphological features between these two groups.

**Fig 1:**
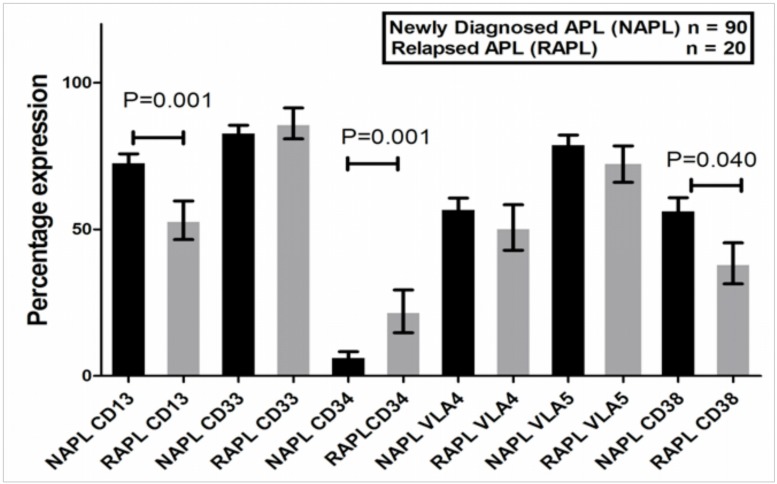
Comparison of bone marrow blast immunophenotypic surface marker expression percentage between newly diagnosed and relapsed patients. The percentage expression of common APL surface markers like CD13, CD33, CD34, CD38, VLA4 and VLA5 are illustrated as grouped bar graphs for newly diagnosed (black bars) and at relapse (grey bars). The mean±SEM were significantly different for CD13, CD34 and CD38 between the newly diagnosed and relapsed patients.

IPT data was available on 90 NAPL and 20 RAPL cases within our study cohort and these were evaluated for this analysis. Fig 1 compares the percentage expression of the common APL markers between NAPL and RAPL. The RAPL had a significantly higher expression of CD34 (P = 0.001) and lower expression of CD13 (P = 0.001) and CD38 (P = 0.040) when compared to the NAPL (Figure B in S1 File—data as median fluorescent intensity).

**Fig 2:**
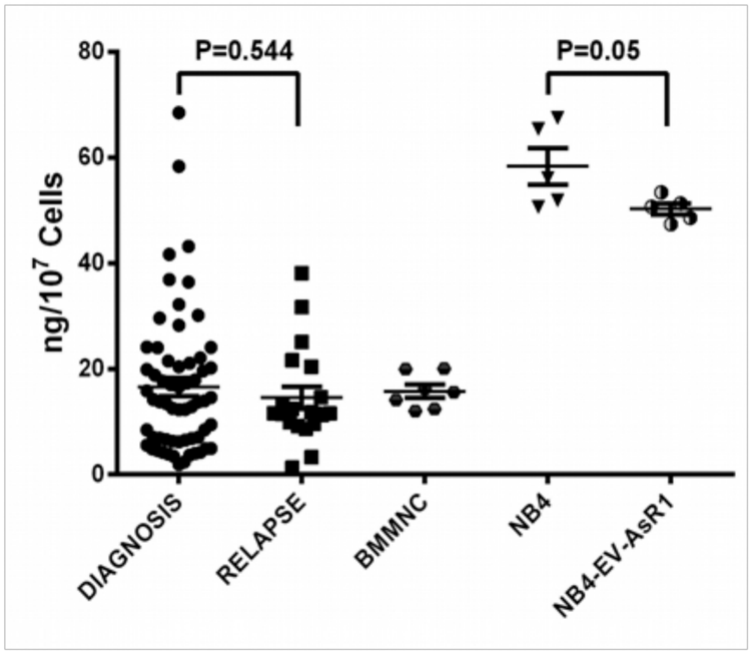
Intracellular arsenic levels in malignant promyelocytes of APL patients at diagnosis, relapse, in normal bone marrow mononuclear cells (BMMNC) and in NB4 cell lines. Scatter plot of the intracellular concentration of arsenic levels in newly diagnosed patients (N = 61) and relapsed patients (N = 19). The level of arsenic is expressed as nanograms (ng) of arsenic detected in 10^7^ cells after 24 hr culture with 0.5uM ATO. Each value was mean of triplicates. There was no significant difference between the median values between the two groups. ATO resistant NB4 cell line NB4-EV-AsR1 (n = 6) had a median ICATO value of 53.7 ng/10^7^ cells, while that of NB4 (n = 5) cells was 65.1 ng/10^7^ cells.

The intracellular arsenic levels (ICATO) were measured in 61 NAPL and 19 RAPL. The ability of both NAPL and RAPL blasts to concentrate ATO intracellular was not significantly different (Median value: 12.85ng/10^7^ cells vs. 11.78ng/10^7^ cells) (Fig 2). The ICATO values did not correlate with clinical parameters such as relapse, EFS or OS.

**Fig 3:**
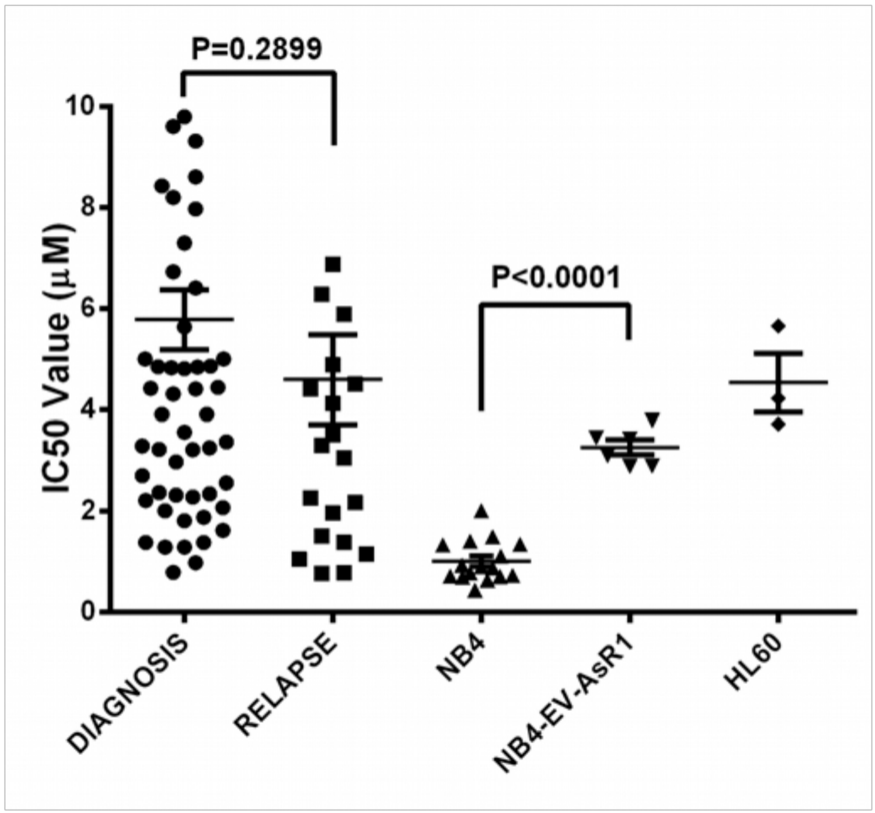
Comparison of *in vitro* cytotoxicity with ATO of NAPL Vs. RAPL and other cell lines. Scatter plots of the IC50 values of newly diagnosed patients (N = 61) and relapsed patients (N = 23) after treating with ATO concentrations ranging from 0.1 to 6 μM for 48 hours is shown. The median IC50 of the newly diagnosed (4.41 μM) and relapse group (3.51 μM) was not significantly different and was higher when compared to that of NB4 cell line (N = 16). The median IC50 of NB4 was 0.9 μM, while that of NB4-EV-AsR1 (ATO resistant NB4 cell line) was 3.25 μM. The IC50 of HL60 was 4.25 μM.

Furthermore *in-vitro* sensitivity of malignant blasts from APL patients at diagnosis and relapse to ATO measured by an MTT assay in 61 NAPL and 23RAPL were not significantly different. The median IC50 value between the two groups was 4.41μM vs. 3.51μM (P = 0.29; Fig 3). These in-vitro values did not correlate with clinical parameters such as relapse, EFS or OS.

### Cytogenetic and genetic analysis

There were both gain and loss of CTG aberrations in RAPL. There was also a trend to an increase number of additional CTG aberrations in the relapsed group. Detailed analysis of the CTG data is given in the supplementary information file (Results section and Table A in S1 File).

As ATO targets the B2 domain of PML and resistance to ATO has been attributed to B2 domain PML gene mutations, we performed Targeted Ion PGM sequencing of the PML B2 domain in 22 out the 26 NAPL cases and in all 26 RAPL cases in whom an adequate library for subsequent sequencing could be obtained. Overall an average of 96.29% genome base coverage was obtained at 100x depth. None of the NAPL cases had any mutations observed in the PML B2 domain. In 4 (15.38%) of RAPL cases at least one mutation was observed in the B2 domain of the PML allele involved in the fusion gene, all of them were novel. The frequencies of the mutations and their function are summarized in Table 2. The absence of mutations was further confirmed by Sanger sequencing.

**Table 2:** Novel mutations found in B2 domain of PML gene in 4 relapsed APL patients detected by the Ion torrent PGM sequencing.

Patient ID—Relapse	Position in chromosome 15 (NC_000015.9)	Type	Zygosity	Genotype	Variation Frequency (%)	Coverage % at 100x	Function	Transcript Variant	Protein Change	IC50 (μM)	ICATO* (ng/10^7^ cells)	Molecular remission achieved post relapse treatment with ATO
SO_2210_PRS19	74315308	SNP	Het	C/T	5.05	99.6%	Nonsense	742C>T	p.Gln248*	1.51	12.39	NA ^+^
SO_2210_PRS45	74315203	SNP	Het	T/A	6.25	99.6%	Missense	637T>A	p.Cys213Ser	2.17	NA	NA^++^
SO_2210_PRS8	74315207	SNP	Het	C/T	93.39	99.6%	Missense	641C>T	p.Ser214Leu	NA	NA	Yes
SO_2210_PRS4	74315222	DEL	Het	AC/A	94.73	99.2%	Frameshift Deletion	-	p.Asp219Glu	6.87	6.93	Yes
	74315224	INS	Het	A/AT	94.15	99.2%	Frameshift Insertion	-	p.Ser220Met			

* ICATO—Intracellular ATO

^+^ Discharged against medical advice

^++^ Died during induction (sepsis)

**Fig 4:**
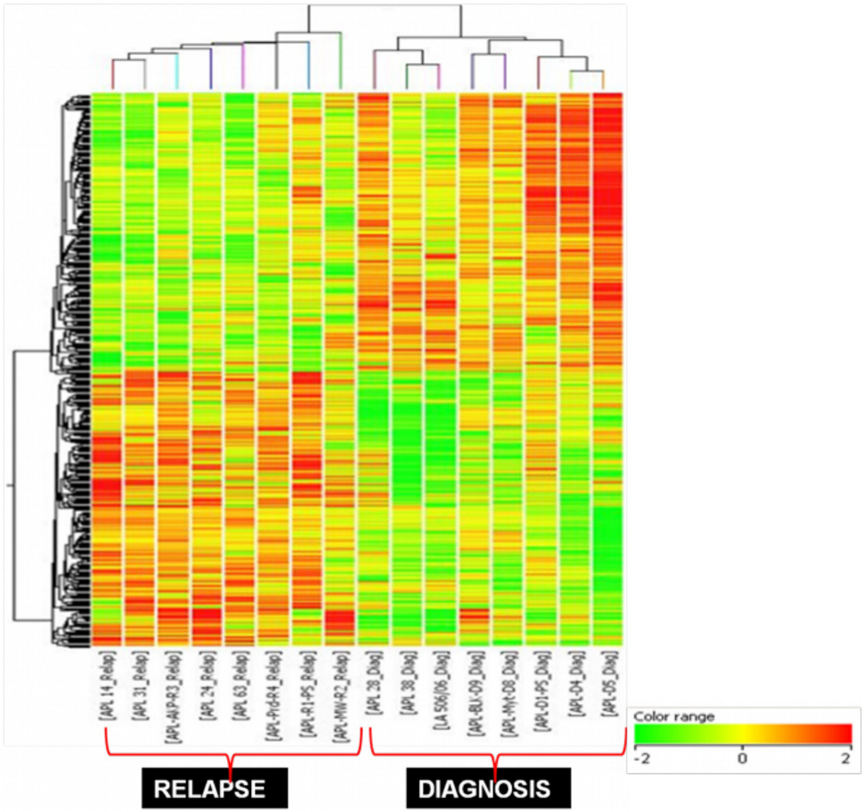
Differential gene expression profile of relapsed Vs. newly diagnosed patients with Acute Promyelocytic Leukemia. The expression profile of eight unmatched relapsed versus newly diagnosed acute promyelocytic leukemia samples. The cluster diagram represents > 2 fold significantly differential gene list of 864 up regulated (red) and 880 down regulated genes (green). The ratios are color coded as indicated in the bar. The complete gene list and annotations are given in the supplementary section (S1 Dataset).

The differential expression profile of 8 NAPL and 8 RAPL were generated. The data was normalized with three different normalization methods (S1 File: Results C) and found to be similar with good correlation. The pattern of differentially expressed genes obtained was robust and similar with all the three normalization strategies. The overview of the gene clustering and the differential expression profile is shown in Fig 4 and in supplementary information (S1 File: additional excel sheet: S1 Dataset). In total, 1744 genes were > 2 fold differentially regulated between the NAPL and the RAPL group which was 8.72% of the 20,000 genes in the array. Of these 864 genes were up regulated while 880 genes were down regulated. The differentially expressed genes were categorized based on biological process, cellular component and molecular function into biological functional groups based on the gene ontologies using Gene Spring and Biointerpretor software (Agilent technologies). Important biological pathways over represented among the differentially expressed genes between the NAPL and RAPL are shown in Table 3 (and Figure C in S1 File).

**Table 3:** Differentially regulated genes in relapsed cases classified into biological functional groups based on the gene ontologies using Gene Spring and Biointerpretor software (Agilent Technologies).

Functional Groups	Total number of genes upregulated	Total number of genes downregulated
Adhesion	71	21
Tight junction	7	1
Stem cell	35	8
Apoptosis	41	11
Immune	50	24
Ion Channel	9	4
Ubiquitin	23	5

The table gives the number of genes and the direction of dysregulation in each of those biological functions in the RAPL compared to the NAPL.

The prominent functional processes differentially regulated in relapsed patients were (i) Cell adhesion: Integrins, Cadherins and Mucins, (ii) Cell survival and anti apoptosis: PI3-AKT, PTEN, NFĸB, MAPK and JAK-STAT, (iii) Stem cell regulation: Wnt, Hedgehog and CD34, (iv) Immune regulation: TNF-receptor super family genes, Interleukins. The complete gene list in each of these pathways is given in the supplementary files (additional excel sheet: S2 Dataset). The heat maps of these functional categories of representative genes in each pathway which are differentially regulated between diagnosis and relapse in APL are shown in Figure D in the S1 File. To validate the reliability of the data, 25 randomly selected genes were analyzed by RQ-PCR and the results were highly concordant with the array data (Figure E in the S1 File).

To verify the reproducibility of the microarray array data obtained from the pilot experiment of 8 unmatched samples at diagnosis and relapse, we did RQ-PCR analysis of the same 25 random genes from the differential regulation list in a fresh set of 10 paired diagnostic and relapse samples. The median fold difference in the expression level of these 25 genes between the paired diagnosis/relapse was compared to that of the differential expression pattern obtained in the microarray. 18/25 genes were differentially regulated in the same direction as predicted by the microarray data (Figure F in the S1 File).

### Comparison of the Effect of Stromal Cell and Malignant Promyelocyte Interaction on the Apoptotic Action of ATO

**Fig 5:**
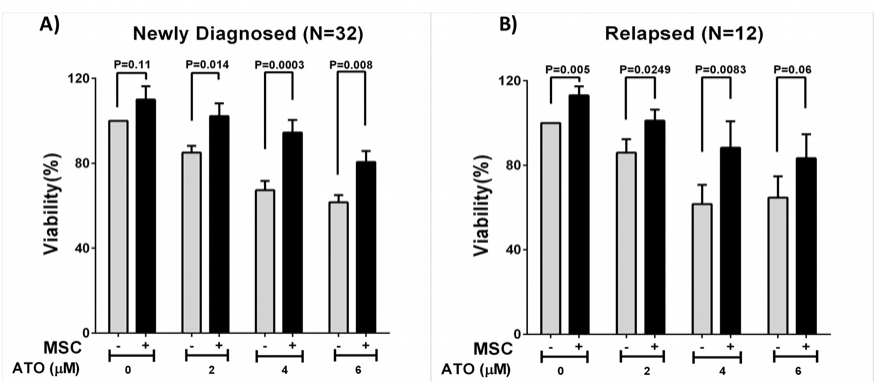
Impact of *in vitro* environment mediated (stromal cell co-culture) interaction on effect of ATO sensitivity at diagnosis and in relapsed cases. The bar graph shows the mean viability percentage of the newly diagnosed [A] and relapsed [B] patients primary cells when cultured with or without stromal cells (MSCs) and at varying concentrations of ATO incubated for 48 hours prior to apoptosis assay. There is a significant protective effect on primary cells in both groups by MSC co-culture to ATO induced apoptosis.

Based on the microarray data and the prominent up-regulation of genes involved in cell adhesion in RAPL compared to NAPL we evaluated the effect of co-culture of primary APL cells from the NAPL and RAPL groups with MSCs and HS-5 (stromal cell line) on ATO sensitivity. We noted a significant protective effect against the apoptotic action of ATO in both groups. The median viability percentage of the NAPL at 4 μM concentration of ATO after 48 hours was 64.34% when grown without MSC which increased to 91.76% when co-cultured with MSCs (N = 32) (0.0003). At the same concentration of 4 μM ATO the median viability percentage of RAPL was 64.81% when grown without MSC, while the median percentage was 96.12% when co-cultured with MSCs (N = 12) (P = 0.024) (Fig 5). The gain in viability was not significantly different between NAPL and RAPL patients studied (median viability gain in co-culture 21.30 Vs. 24.18 respectively; P = 0.96). A similar protective effect was not seen when malignant promyelocytes were co-cultured with HUVEC cell lines (data not shown).

Similar to the APL primary cells, the protective effect was observed in the co-culture of NB4 cells with MSCs and HS-5 (n = 8; P = 0.02) (Figure G in the S1 File). Blocking antibodies against VLA-4 and VLA-5 did not affect the protective effect of stromal cells on NB4 cells (data not shown). There were no significant changes in the surface marker expression of the VLA4, VLA5, CD184, CD44, CD123 and CD34 in either primary cells or NB4 cells on co-culture with HS-5 cells for 48 hours. The proliferation assay using CFSE staining in the presence of HS-5 showed a significant reduction in the proliferation of NB4 cells in co-culture when compared to NB4 cells alone at 48 and 72 Hrs respectively (N = 3; P = 0.003 at 48H) (Figure H in the S1 File). Cell cycle analysis at 48 hours co-culture of NB4 with HS-5 cell line showed that there was a significant increase in the percentage of G0/G1 cells from 48% to 62% (n = 6; P = 0.004) on co-culture when compared to the NB4 cells alone (Figure H in the S1 File).

## Discussion

While ATO is effective in inducing molecular remissions in the majority of patients with RAPL the risk of relapse is high especially in the absence of consolidation with an autologous SCT[13]. Having noted the inferior clinical response in patients with RAPL in comparison to NAPL we wanted to evaluate the potential mechanisms to explain this difference.

Recent interest has focused on mutations in the oncogenic PML-RARA gene, especially mutations in the B2domian of the PML component, which can result in acquired resistance to ATO[18]. However, majority of patients who do relapse after treatment with ATO based regimens do not have such mutations[19] and additionally primary or secondary drug resistance to ATO is rare in the clinic. The majority of RAPL patients achieve hematological and molecular remissions even after multiple relapses on treatment with ATO though the duration of response decreases and the risk of disease recurrence increase with each such relapse. Clonal evolution with mutations leading to ATO resistance cannot explain the observations seen in the clinic in the majority of patients.

At the time of diagnosis it was interesting to note that relapsed patients had a significantly lower WBC count and significantly higher platelet count. It was also of interest to note that in this prospective study there were a significantly lower number of IC bleeds and lower utilization of platelet concentrates during induction therapy in relapsed cases suggesting that coagulopathy was probably less severe in this group.

There was earlier data to suggest that malignant promyelocytes were able to better concentrate ATO intracellularly than other malignant cells[24] and recent studies to suggest that there were ABC transporters involved in ATO efflux[25]. We had hypothesized that in relapsed patients such efflux mechanisms were probably involved and contributed to the increased relapse in these patients. However, in this study we could not demonstrate any difference in the ability to concentrate ATO intracellularly between newly diagnosed and relapsed cases nor could we find any association with these values and subsequent relapses. Consistent with these observations the GEP data did not demonstrate differential expression of any of the known transporters involved with ATO in these two cohorts. Similarly there was no difference in the *in-vitro* sensitivity of malignant promyelocytes to ATO in both groups and no correlation with subsequent clinical events. This data is consistent with the clinical observation that primary or secondary resistance to ATO is very rare in patients. The previously reported PML B2 domain A216V ATO resistant mutation[18] was not detected in any of the patients in this series. Of the 4 patients in this series that had PML B2 domain mutations two achieved molecular remission with ATO based regimens. Of the remaining two, one patient died in induction and one patient was discharged against medical advice however even in these two patients the in-vitro IC-50 data suggests that the blasts were sensitive to ATO (IC50 below median value).

The GEP pattern of increased expression early HSC markers such as CD34 and decreased expression of mature markers such as CD13, CD38 and CD44 in the relapsed group along with increased expression of other genes in the stem cell pathway (n = 51) suggests that there could potentially be a shift to a more immature phenotype and a potential expansion in the leukemia initiating compartment in the RAPL patients. The IPT data validates GEP data by demonstrating a similar increased CD34 and reduced CD38 expression in RAPL. While there is limited data on the phenotype of the stem cell / leukemia initiating compartment in human APL there is some data to suggest that this population, at least in a mouse model, resides in promyelocytes that are CD34(+), c-kit(+)and FcgammaRIII/II(+)[26, 27].

Based on the prominent up regulation in the GEP of adhesion and cytokine genes / pathways, previously reported to be involved in micro environment mediated drug resistance (EM-DR) in other malignancies, in the RAPL cohort we further evaluated this with in-vitro experiments. This is the first study to demonstrate innate EM-DR to ATO in APL leukemic cells which is seen in both cell lines and primary cells. This innate EM-DR was marginally more prominent in relapsed cases though this did not achieve statistically significant difference. The exact mediators and mechanism needs further evaluation but our preliminary data suggests that the co-culture induces quiescence that could potentially contribute to ATO resistance.

Though there are previous studies reporting GEP of APL patients at diagnosis, there are none reporting the molecular gene expression signature of patients at the time of relapse following treatment with ATO. Even though we had a limitation of comparing unmatched samples at diagnosis and at relapse, the fact that the expression signature was subsequently validated in a fresh cohort of 10 paired diagnosis/relapse patient samples gives us the confidence to pursue the pathways and genes picked up by microarray study to understand the disease progression. The major pathways and genes that are differentially expressed in relapsed cases and the potential role played by them in relapse is summarized in the supplementary section.

From this data we conclude that recurrent relapse in RAPL to ATO based therapy is probably multi-factorial. In APL clonal evolution resulting in by acquisition of mutations leading to ATO resistance (such as the recently reported PML B2 domain mutation) are rare as is demonstrated by the few cases in which this has been reported [18, 19] and the ability of ATO to induce molecular remission in the majority of patients with multiple relapses even after prior exposure to ATO. Based on our observations we hypothesize that contributory factors to relapse in APL following treatment with ATO could include the expansion of the leukemia initiating compartment and an increase in EM-DR to ATO (Figure I in the S1 File). Both these mechanisms need further evaluation and additional experiments to validate these findings and understand their molecular basis.

The microarray data discussed in this manuscript have been deposited in the NCBI Gene Expression Omnibus (GEO) under the GEO series accession number GSE42030 and GSE42031.

## Supporting Information

S1 Dataset Excel sheet with a list of genes that were differentially expressed when comparing newly diagnosed and relapsed patient samples. All genes with > 2 fold differential expression in relapsed patients samples in comparison to newly diagnosed cases are included in this list.
(XLSX)

S2 Dataset Excel sheet with complete gene list arranged according to pathways that differentially regulated. Complete gene list in the pathways that were differentially regulated in relapse in comparison to newly diagnosed APL patients on a microarray gene expression profile.
(XLSX)

S1 File Figure A. *In vitro* cytotoxicity of cell lines and primary cells to ATO. The mean IC50 of HL60 cell line was 4.25uM (which is equivalent to the log concentration of ATO of 3.6 as represented in the above graph) [B]. There was negligible cell kill of the other cells checked like the PBMNCs [A], MSCs [C] and U937 [D] at the concentrations of ATO used in this study. All the experiments were reported as an average of at least 3 independent experiments. Figure B. Comparison of the Median Fluorescent Intensity of bone marrow blast immunophenotype markers in newly diagnosed vs. relapsed patients. The median fluorescent intensity (MFI) values of the immunophenotypic markers of the patients in the newly diagnosed and relapsed group are represented as individual stick bars. Figure C. Differentially regulated genes in relapsed cases classified into biological functional groups based on the gene ontologies using Gene Spring and Biointerpretor software. The genes differentially up regulated [A] and down regulated [B] in relapse group when compared to newly diagnosed group are fit into biological functions based on their gene ontologies using the Biointerpretor software (Agilent Technologies). [C] The table gives the number of genes and the direction of dysregulation in each of those biological functions. Figure D. Heat maps of differentially regulated genes in functional pathways. Heat map demonstrating the genes up regulated (red) and down regulated (green) at least 2 fold in relapse (REL) in comparison to at diagnosis (DX) classified in functional categories. The ratios are color coded as indicated in the bar. Figure E. Validation of the gene expression differential regulation using RQ-PCR- ΔΔCT method. Comparison of RQ-PCR data of 25 genes from the same set of samples from which the microarray data was derived. Median fold difference was calculated by ΔΔCT method. Individual cases values are average of triplicates. Concordant results were obtained in all but four genes as illustrated. Figure F. Validation of a set of microarray predicted gene expression in a second cohort of paired matched samples (n = 10) that were available both at diagnosis and at relapse. The bar graph shows the comparison between the relative fold difference in the selected 25 genes between the initial diagnosis and at the subsequent relapse of 10 patients. 18 of the 25 genes evaluated by RQ-PCR in these cases had the regulation altered in the same direction as predicted by the microarray data from the initial cohort. Figure G. Protective effect of stromal cell co-culture of NB4 cells against ATO. The bar graph shows the mean viability percentage of the NB4 cells when cultured with or without stromal cells (MSCs) and at varying concentrations of ATO incubated for 48 hours prior to apoptosis assay. There is a significant protective effect in NB4 cells by MSC co-culture to ATO induced apoptosis (N = 9). Figure H. Effect of stromal cell interaction on cell cycle and cell proliferation assay on NB4 cells in the stromal cell co-culture system. [A] Cell cycle analysis NB4 cells with and without co-culture with HS-5 cells for 48H stained with PI [B]. The NB4 cells were stained with CFSE and cultured with and without HS-5 cells, for 24, 48 and 72 hours respectively. Figure I. Model of ATO resistance based on our observations in this study.
(DOCX)
