# Supplementary material for: Comparison of Newly Diagnosed and Relapsed Patients with Acute Promyelocytic Leukemia Treated with Arsenic Trioxide: Insight into Mechanisms of Resistance
Source: PLoS One. 2015 Mar 30;10(3):e0121912. doi: 10.1371/journal.pone.0121912 (PMC4378855; doi:10.1371/journal.pone.0121912)
Supplement: S1 File — The mean IC50 of HL60 cell line was 4.25uM (which is equivalent to the log concentration of ATO of 3.6 as represented in the above graph) [B]. There was negligible cell kill of the other cells checked like the PBMNCs [A], MSCs [C] and U937 [D] at the concentrations of ATO used in this study. All the experiments were reported as an average of at least 3 independent experiments. Figure B. Comparison of the Median Fluorescent Intensity of bone marrow blast immunophenotype markers in newly diagnosed vs. relapsed patients. The median fluorescent intensity (MFI) values of the immunophenotypic markers of the patients in the newly diagnosed and relapsed group are represented as individual stick bars. Figure C. Differentially regulated genes in relapsed cases classified into biological functional groups based on the gene ontologies using Gene Spring and Biointerpretor software. The genes differentially up regulated [A] and down regulated [B] in relapse group when compared to newly diagnosed group are fit into biological functions based on their gene ontologies using the Biointerpretor software (Agilent Technologies). [C] The table gives the number of genes and the direction of dysregulation in each of those biological functions. Figure D. Heat maps of differentially regulated genes in functional pathways. Heat map demonstrating the genes up regulated (red) and down regulated (green) at least 2 fold in relapse (REL) in comparison to at diagnosis (DX) classified in functional categories. The ratios are color coded as indicated in the bar. Figure E. Validation of the gene expression differential regulation using RQ-PCR- ΔΔCT method. Comparison of RQ-PCR data of 25 genes from the same set of samples from which the microarray data was derived. Median fold difference was calculated by ΔΔCT method. Individual cases values are average of triplicates. Concordant results were obtained in all but four genes as illustrated. Figure F. Validation of a set of microarray predicted gene expr [file pone.0121912.s003.docx]

**Supplementary Information**

**Comparison of Newly Diagnosed and Relapsed Patients with Acute Promyelocytic Leukemia treated with Arsenic trioxide: Insight into Mechanisms of Resistance to Arsenic Trioxide**

Ezhilarasi Chendamarai^1^, Saravanan Ganesan^1^, Ansu Abu Alex^1^, Vandana Kamath^2^, Sukesh C. Nair^2^, Arun Jose Nellickal^3^, Nancy Beryl Janet^1^, Vivi Srivastava^4^, Kavitha M. Lakshmi^1^, Auro Viswabandya^1^, Aby Abraham^1^, Mohammed Aiyaz^5^, Nandita Mullapudi^5^, Raja Mugasimangalam^5^, Rose Ann Padua^6^, Christine Chomienne^6^, Mammen Chandy^1*^, Alok Srivastava^1^, Biju George^1^, Poonkuzhali Balasubramanian^1^, Vikram Mathews^1^

**SI File: Materials and Methods**

**Methods A: Morphological Analysis** Bone marrow slides of all newly diagnosed and relapsed APL patients were stained using a Wright Giemsa stain. Slides were reviewed by two independent and experienced hematopathologists.

**Methods B: Immunophenotypic Analysis** Immunophenotyping (IPT) was done on bone marrow samples collected from newly diagnosed and relapsed APL patients. Briefly cells were labeled using a panel of monoclonal antibodies to CD13, CD33, CD34, CD19, CD38, CD49d, CD49e, CD184, CD45 and HLADR directly conjugated with FITC, PE, PerCP or APC after red cell lysis using ammonium chloride and were incubated for 20 minutes. The cells were then washed and analyzed using FACSCalibur (Becton Dickenson, Mansfield, MA). Gating strategy to characterize the blast population consisted of using a CD45 Vs. SSC to define the CD45 dim blast population and all subsequent analysis of markers was restricted to this blast population. Data analysis was performed using CellQuestPro software (Becton Dickenson).

**Methods C: Cytogenetical Analysis** All cytogenetic studies were performed at the Cytogenetic unit of our institution according to standard well established protocols with the karyotypes designated according to the International System for Human Cytogenetic Nomenclature (ISCN) guidelines(1). Images were analyzed using Ikaros software (Metasystems, Altlussheim, Germany).

**Methods D: Intracellular arsenic measurement**  The intracellular level of arsenic was measured using well established and published protocols(2, 3). Briefly, 2 x 10^7^ cells were washed and suspended in RPMI media with 0.5 µM concentration of ATO and incubated for 24 hours. The cell pellets were then washed twice in Ca^2+^ and Mg^2+^ free PBS (Stem Cell Technologies, Vancouver, Canada). The cell pellets were digested with a standard volume of suprapur nitric acid and hydrogen peroxide (Merck, Darmstadt, Germany) (2:1 v/v) at 45^0^C for 16 hours. An aliquot of this final solution was analyzed using Atomic Absorption Spectrometer with EDL support equipment (Perkin Elmer, Massachusetts, USA) in the Clinical Biochemistry Department of our institution. All the standardizations were carried out in the Clinical Biochemistry department of our institution. The linear range of arsenic measurement by AAS was estimated to be between 1 ng/ml to 50ng/ml. The patient and cell line samples assessed for intracellular arsenic concentration had an average level of 20ng/ml which was above the sensitivity threshold of this method and in the linear detection range. The inter assay and intra assay variability of this method had a coefficient of variation (CV) in the range of 0.97% to 7.38% and 1.29% to 7.96% respectively for the evaluated concentration range of 100 to 1 ng/ml.

**Methods E: *In vitro* cytotoxicity of ATO**  An *in vitro* ATO sensitivity assay of malignant cell lines and primary APL cells was standardized using an MTT assay system (Biotium, Inc. CA, USA). MTT assay was read on an EL800 Elisa reader (BioTek India, Mumbai, India) and the IC50 generated using Graph Pad Prism software. MTT assay was also done on normal mesenchymal stromal cells (MSC), normal PBMNC, non-APL myeloid cell lines HL60 and U937. NB4 cell line was used to standardize the variables such as the optimal number of cells, optimal hours of incubation needed for the assay. A linear relation between the cell number and the optical density was seen at 10^5^ cells per well and the optimal cell kill was obtained at 48 hours of incubation of ATO. The assay was reproducible and comparable with the published IC50 value of NB4 cell lines with median IC50 of 0.88µM and the mean ± SD of 10 independent experiments was 1.004±0.4.

***Comparison of in vitro sensitivity of NB4 to ATO with other cell type*** MTT assay was done for the mesenchymal stromal cells (MSCs), peripheral blood mononuclear cells (PBMNCs) and myeloid cell lines HL60 and U937. There was insufficient kill of the normal MSCs, PBMNCs, and that of the U937 cell lines at the concentration studied here as shown in the figure A. The HL60 had a median IC50 value of 4.25µM±1.0 ATO.

**
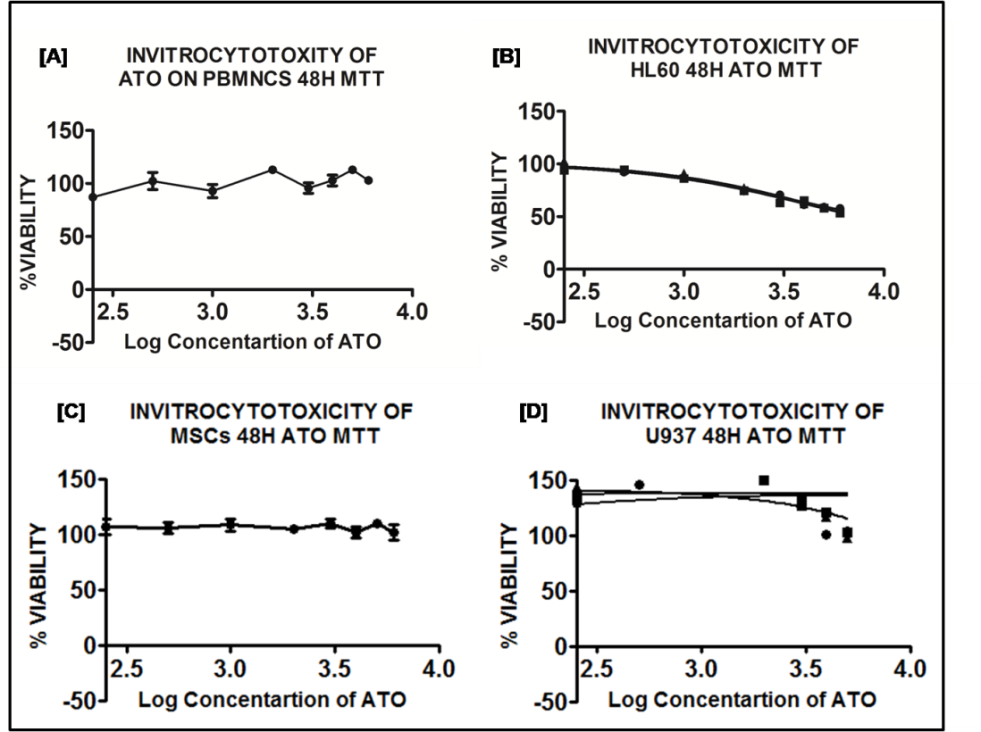
**

**Figure A: *In vitro* cytotoxicity of other cell lines and primary cells to ATO** The mean IC50 of HL60 cell line was 4.25uM (which is equivalent to the log concentration of ATO of 3.6 as represented in the above graph) [B]. There was negligible cell kill of the other cells checked like the PBMNCs [A], MSCs [C] and U937 [D] at the concentrations of ATO used in this study. All the experiments were reported as an average of at least 3 independent experiments.

**Methods F: RNA extraction, cDNA synthesis, library preparation and sequencing by Ion torrent Personal Genome Machine (PGM):**

Total RNA was extracted using trizol (Invitrogen, CA, USA) from 27 NAPL and 25 RAPL cases. cDNA was synthesized using SuperScript II reverse transcriptase (Invitrogen) using standard protocol. Library preparation for Ion PGM sequencing were performed following certified protocols (Life-technologies, Carlsbad, CA, USA). Amplicons were generated with the starting amount of 5ul of cDNA and 50 cycles of PCR was performed to amplify the region of PML-RARA coding sequence using specific primers (given below). 2ul of amplified samples was used for a second round of PCR to amplify the B2 domain of PML region (300bp). 1ul of round 2 products was used for next round of PCR (round 3), 10cycles of PCR was performed, and Index barcodes were added using modified primers which had adaptor sequences. Equal amount of barcoded amplicon was pooled and cleaned using Agencourt Ampure XP SPRI beads (Beckman Coulter). They were quantified using Qubit, pooled in equimolar ratios and validated for quality by running an aliquot on High Sensitivity Bioanalyzer Chip (Agilent technologies, Singapore) and proceeded with the Ion PGM sequencing. Post trimming the reads was aligned to the reference PML amplicon using TMAP algorithm and variants were detected by the plugin Variant caller (v) of Torrent Suite v3.6.2, using high stringency settings for calling somatic variants.

**Primers used for the target amplification**: Forward primer: TTCCTGGACGGCACCC, Round 1 reverse primer: TCTACCCGCATCTACAAGC; Round 2 reverse primer: GCGCCAAAGGCACTATCC.

**Methods G: Microarray Gene expression Profiling:**

The microarray processing was done at the Genotypic Technologies, Bengaluru, India.

**Cell line samples:** In addition to patient samples (details in main manuscript) naïve NB4 cells and the ATO resistant clone (NB4-EV-AsR1) cells were cultured in RPMI media with 10% FBS. The NB4-EV-AsR1cells were grown without ATO for one week prior to this experiment. Two independent flask of these two cell lines were maintained to serve as biological replicates for the microarray experiment. The naïve NB4 cells was considered as control and the gene expression profile of resistant clone was compared and contrasted with the control and a differential gene expression profile was generated.

***RNA extraction:***2x10^7^ cells were re-suspended in RNA later solution (Qiagen GmbH, Hilden, Germany) and all the samples were stored at -20^0^C storage until shipped to the microarray facility (Genotypic Technology, Bengaluru). Total RNA was extracted from the leukocytes using the Qiagen RNeasy mini kit. Total RNA integrity was assessed using RNA 6000 Nano Lab Chip on the 2100 Bioanalyzer (Agilent, Palo Alto, CA). Total RNA purity was assessed by the NanoDrop® ND-1000 UV-Vis Spectrophotometer (Nanodrop technologies, Rockland, USA).

***Labeling, hybridization and scanning:***The samples were labeled using Agilent Quick-Amp labeling Kit (p/n5190-0442). The labeled cRNA samples were hybridized on to a Agilent Human Whole Genome 4x44k Gene Expression Array ( AMADID: 14850) . Fragmentation of labeled cRNA and hybridization were done using the Gene Expression Hybridization kit of Agilent (Part Number 5188-5242). Hybridization was carried out in Agilent’s Surehyb Chambers at 65º C for 16 hours. The hybridized slides were scanned using the Agilent Microarray Scanner G2505C at 5 micron resolution.

***Microarray Data Analysis:*** Data extraction from Images was done using Agilent Feature Extraction software v 10.5.1.1 Feature extracted data was analyzed using GeneSpring GX version 11 software from Agilent. Normalization of the data was done in GeneSpring GX using the 75^th^ percentile shift and Normalization to Specific Samples. Samples were grouped based on the Relapse and Diagnosis. Significant genes up and down regulated between Relapse and Diagnosis were identified. Student T-test p-value was calculated using volcano Plot Algorithm using Genespring GX Software. Differentially regulated genes were clustered using hierarchical clustering to identify significant gene expression patterns Genes were classified based on functions and pathways using Genotypic Technology Private Limited-Biointerpreter**-**Biological Analysis Software.

**Methods H: The role of microenvironment interactions mediated resistance against ATO *in vitro***

**Immunophenotyping:** IPT of NB4 cells in co-culture with HS-5 cells for 48 hours was done for the surface markers such as CD49d (VLA4), CD49e (VLA5), CD184, CD44, CD123 and CD34 as mentioned in the supplementary methods section 2.

**Cell cycle analysis:** The NB4 cells with and without co-culture with HS-5 cells for 48H were harvested. Around 1x10^6^ cells were washed twice with PBS. 1ml of cell cycle buffer was added to the cells and incubated in the dark for 10 minutes. The FL2 voltage was adjusted so that the first peak comes to 200 and at least 10000 cells was acquired by flow cytometry. Analysis for Cell cycle was done using FlowJo software (Treestar, Inc., OR, USA).

**Cell proliferation assay**: The NB4 cells were stained with CFSE (Cell Technologies, CA, USA) at 25x concentration for 30 minutes. After washing with PBS the cells were cultured with and without HS-5 cells. The cells (10^6^) were harvested at 24, 48 and 72 hours respectively to assay for proliferation in FL-1 channel using FACScalibur.

**Blocking antibodies to adhesion molecules:** The NB4 cells were incubated with the blocking antibodies to integrins like VLA-4 and VLA-5. These cells were added (1x10^5^cells/well) on a layer of MSC in 24 well plate or alternatively in 24 well fibronectin coated plate. Appropriate controls were included such as, control wells with NB4 without MSCs, control wells with MSC without NB4, NB4 + MSC – without antibodies, NB4 + MSC with Isotypic antibody control. After 2 hour culture of these cells with MSCs or on fibronectin coated plate, the cells were exposed to 2µM, 4µM and 6µM ATO. Post 48 hour incubation the sensitivity to ATO was assessed by Annexin V apoptosis assay.

**SI Results**

**Results A: Comparison of IPT of NAPL Vs. RAPL** CD34 value of >20% was seen in 7/90 (7.7%) of newly diagnosed and 7/20 (35%) of relapsed. Of the cases analyzed, paired diagnosis and relapse samples were available for three cases and in them an increased percentage expression at relapse was observed (75.5%, 64.40% and 27.6%) from almost negative expression at diagnosis (2.7%, 2.3% and 1% respectively).

**Figure B: Comparison of the Median Fluorescent Intensity of immunophenotype markers in newly diagnosed vs. relapse.** The median fluorescent intensity (MFI) values of the immunophenotypic markers of the patients in the newly diagnosed and relapsed group are represented as individual stick bars. The P-Values were generated using student’s t-Test.

**
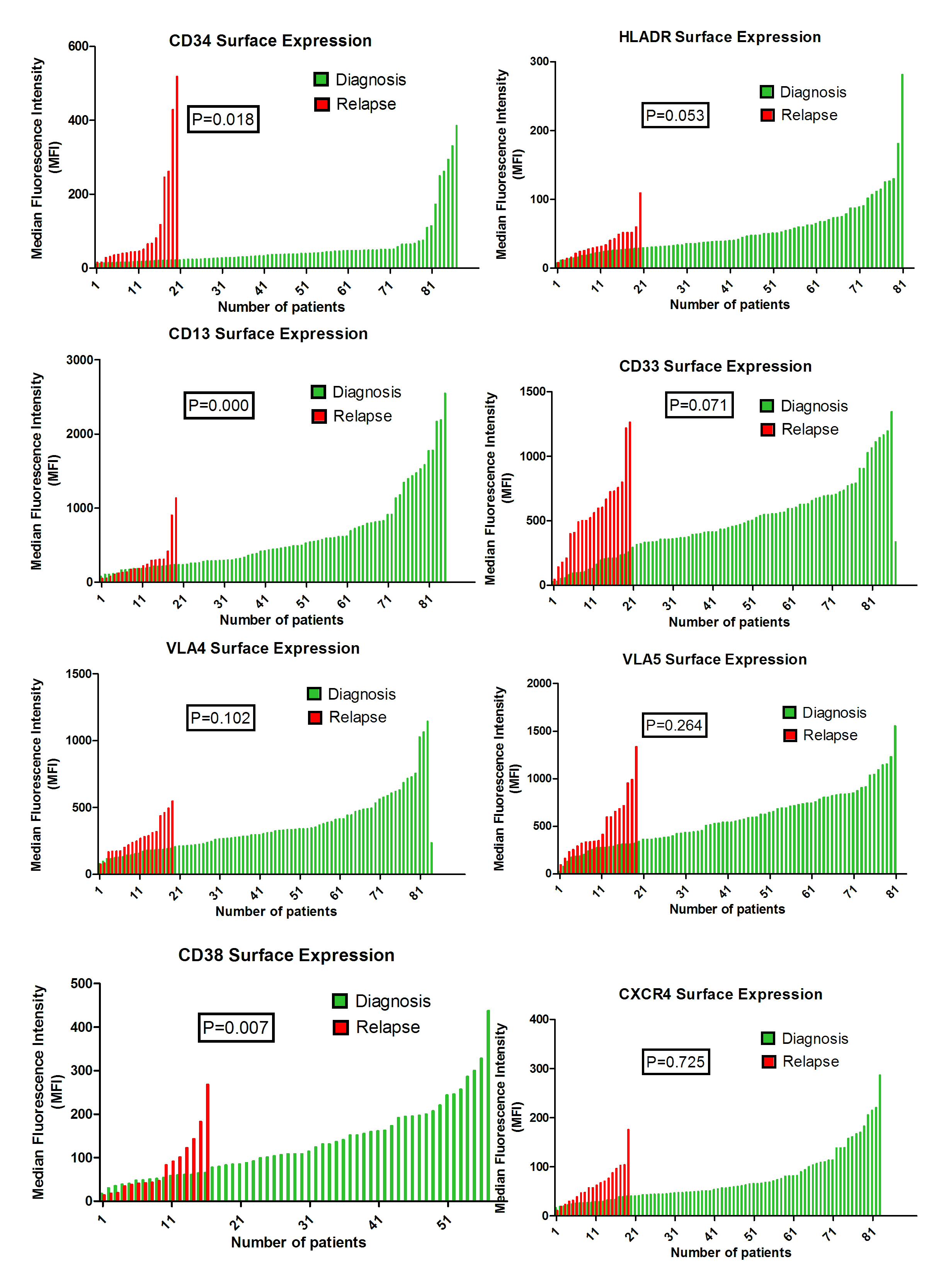
**

**Results B: Comparison of Cytogenetics data findings in newly diagnosed and relapsed APL patients :** Thirty three out of the 92 NAPL (35.86%) had additional CTG abnormalities while 12/19 RAPL (63.15%) had an additional cytogenetic abnormalities (P = 0.06). Complex karyotypes(1) were seen in 24/92 (26.08%) at diagnosis Vs. 7/19 (36.84%) in the relapsed group (P=0.34). CTG data at both diagnosis and subsequent relapse was available in 10 cases and is summarized in table A.

**Table A: CTG aberrations in 11 paired newly diagnosed and relapsed patients where CTG data was available at diagnosis and at relapse(s).**

| **S.No.** | **Initial Diagnosis** | **Relapse** | |
| --- | --- | --- | --- |
|  |  | **First** | **Second** |
| 1 | 47,XY, +8,  t(15;17)(q22;q21) [5] / 48,idem,+21 [5] | 48,XY,+8,t(15;17)(q22;q21),  +21[16] | 48,XY,+8,t(15;17)(q22;q21),  +21[13]/***49,idem,+10***[5] |
| 2 | ND | 46,XY,t(3;9)(p21;q21),  ins(15;17)(q22;q21)[10]/46,XY[2] | 46,XY,t(3;9)(p21;q31),  ins(15;17)(q22;q21)[17]/46,XY[3] |
| 3 | 46,XY,t(15;17)(q22;q21)[18]/**46,idem,add(21)**(q22)[3] | 46,XY,**add(8)**(q24.3),  t(15;17)(q22;q21)[20] | 46,XY,**add(8)(**q24.3),  t(15;17)(q22;q21)[20] |
| 4 | 46,XY,t(15;17)(q22;q21)[8]/ 46,XY[12] | 47,XY,**add(7)(**q36),t(15;17)(q22;q21**),**  **+21**[4]/46~47,**idem,add(15**)(p11.2),-**16,-21,+1~2mar**[cp12]/46,XY[4] | NA |
| 5 | 46,XY,t(15;17)(q22;q21)[12]/47,idem**,+mar?+21 or der(21)**[8] | 47,XY**,+8**,  t(15;17)(q22;q21) [14]/46,XY[4] | NA |
| 6 | 46,XY,t(15;17)(q22;q21)[19] | 46,XY,t(15;17)(q22;q21)[20] | NA |
| 7 | 46,XY,t(15;17)(q22;q21)[3]/  **46,idem,del(9**)(q21q31)[12] | 46,XY,t(15;17)(q22;q21)[20]/  46,XY[5] | NA |
| 8 | 46,XY,t(15;17)(q22;q21)[14]/46,XY[6] | 46,XY,t(15;17)(q22;q21)[10]/  46,XY[10] | NA |
| 9 | 46,XY,t(15;17)(q22;q21)[15]/46,XY[5] | 46,XY,t(15;17)(q22;q21)[20] | NA |
| 10 | 46,XY,t(15;17)(q22;q21) [4] / **47,idem,+mar** [3] / **48,idem,+mar x 2** [12] | 48,XY,t(15;17)(q22;q21**),**  **+mar der(Y),+mar?der(18)[**16]/  46,XY[4] | NA |
| 11 | 46,XY,t(15;17)(q22;q21)[18] | 46,XY,t(15;17)(q22;q21)[20] | NA |

ND- not done; NA-not applicable

The aberrations highlighted with bold in the initial diagnosis column were lost at the time of relapse; the aberrations underlined in the first relapse column were detected at relapse which is not found at diagnosis; new changes at second relapse are italicized in the second relapse column.

**Result C: Gene expression profile** The differential expression profile of 8 newly diagnosed and 8 relapsed patients were generated. In two of these 16 cases (both newly diagnosed) enrichment process was required to reach >80% blasts + promyelocytes. The data normalized with three different normalization methods (75th Percentile Shift Normalization, Quantile normalization and Scale Normalization method) was found to be similar with good correlation. The pattern of differentially expressed genes obtained was robust and similar with all the three normalization strategies.

**Figure C**: **Differentially regulated genes in relapsed cases classified into biological functional groups based on the gene ontologies using Gene Spring and Biointerpretor software.** The genes differentially up regulated [A] and down regulated [B] in relapse group when compared to newly diagnosed group are fit into biological functions based on their gene ontologies using the Biointerpretor software (Agilent Technologies). [C] The table gives the number of genes and the direction of dysregulation in each of those biological functions.

**[A]** **[B]**


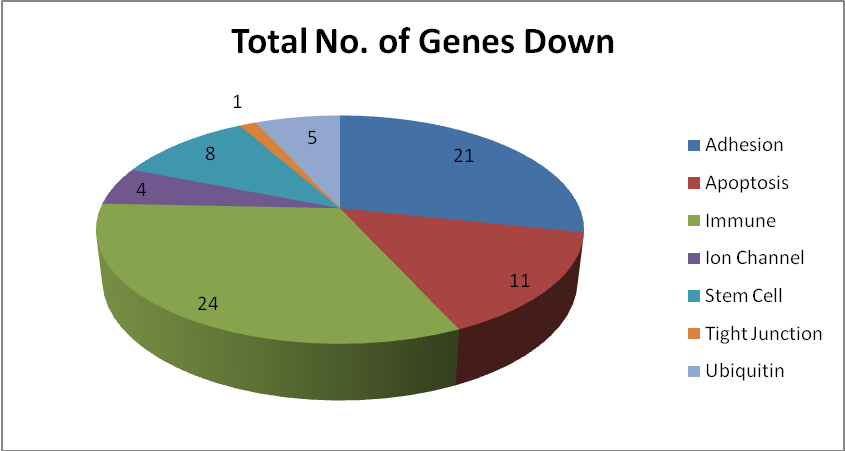

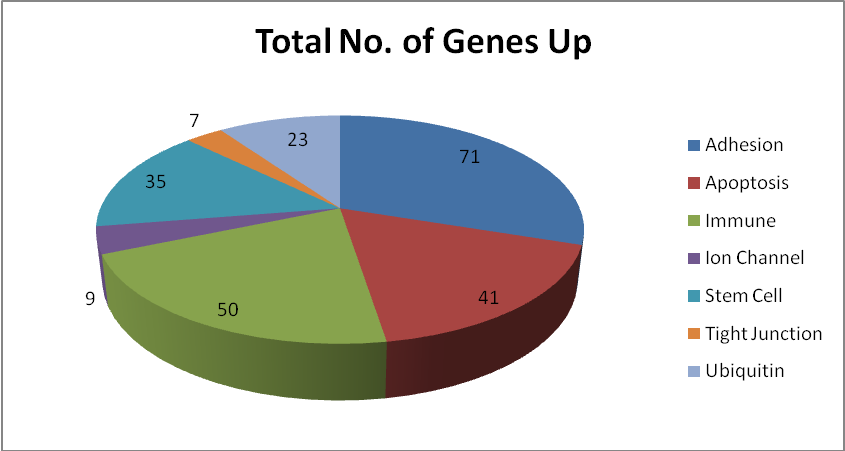


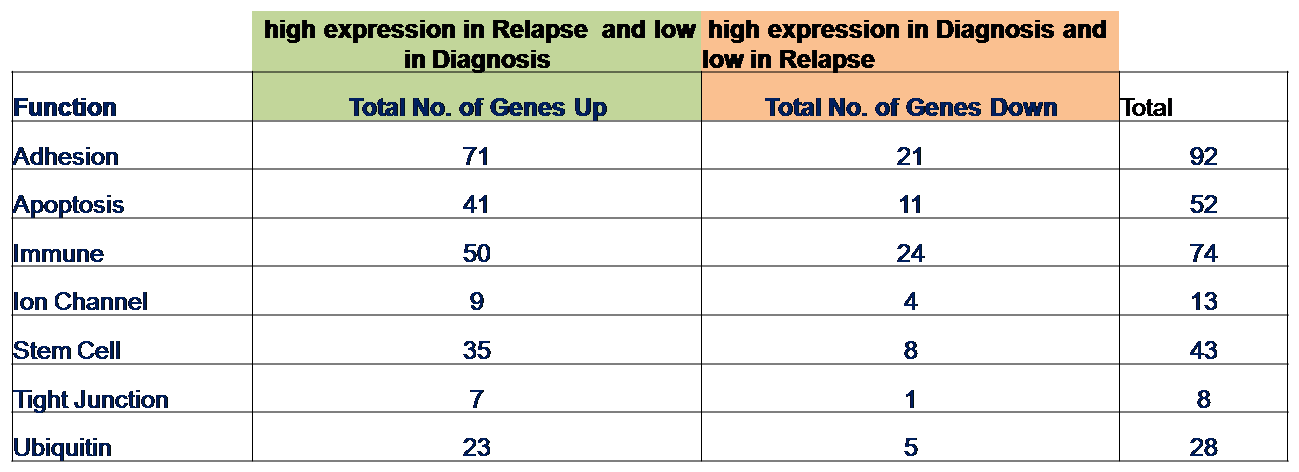
**[C]**

**Figure D: Heat maps of differentially regulated genes in functional pathways**. Heat map demonstrating the genes up regulated (red) and down regulated (green) at least 2 fold in relapse (REL) in comparison to at diagnosis (DX) classified in functional categories. The ratios are color coded as indicated in the bar. (For expansion of the complete gene list and annotations see: S_Dataset 2.xlsx).


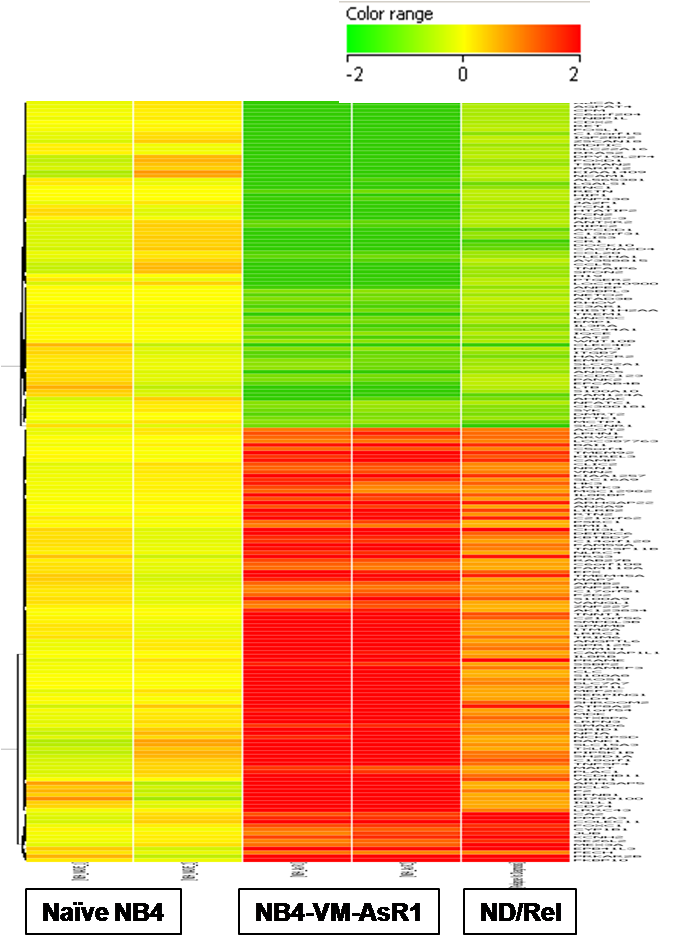

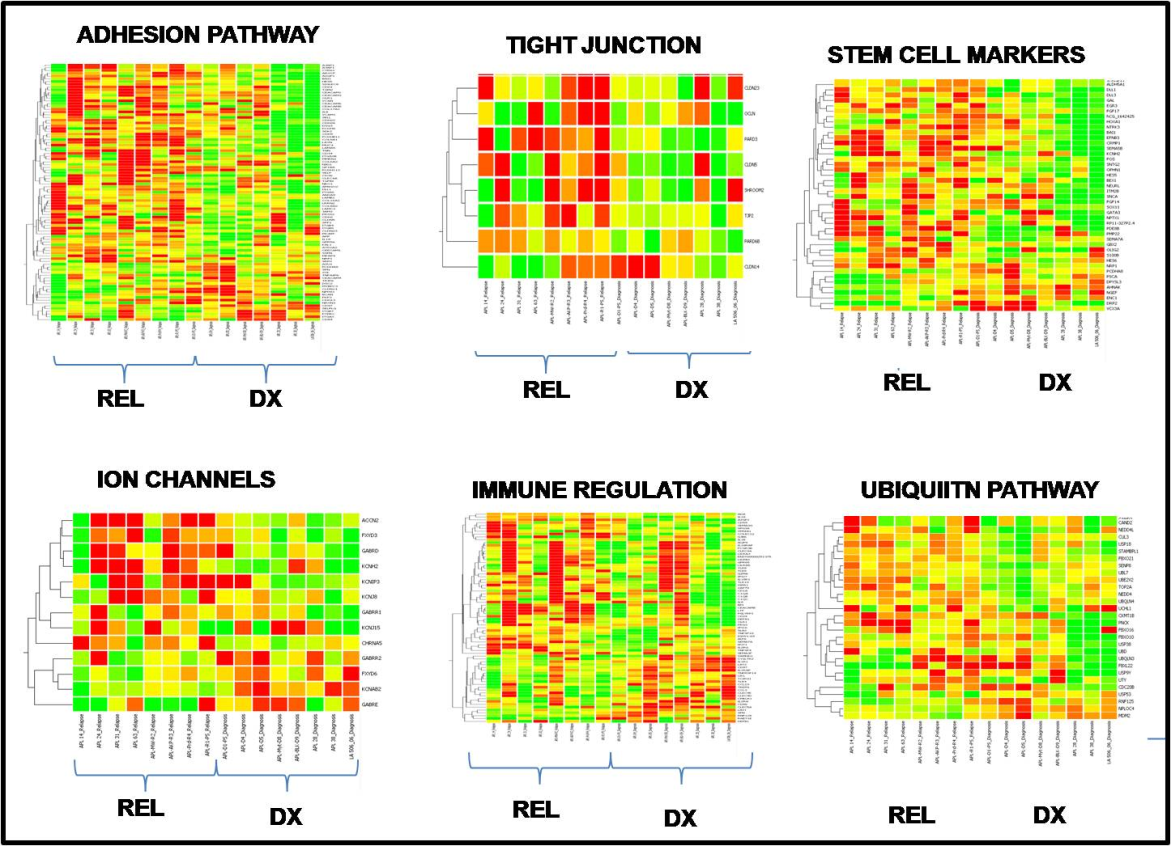


**Figure E**: **Validation of the gene expression differential regulation using RQ-PCR -** **ΔΔCT method.** Comparison of RQ-PCR data of 25 genes from the same set of samples from which the microarray data was derived. Median fold difference was calculated by ΔΔCT method. Individual cases values are average of triplicates. Concordant results were obtained in all but four genes as illustrated.


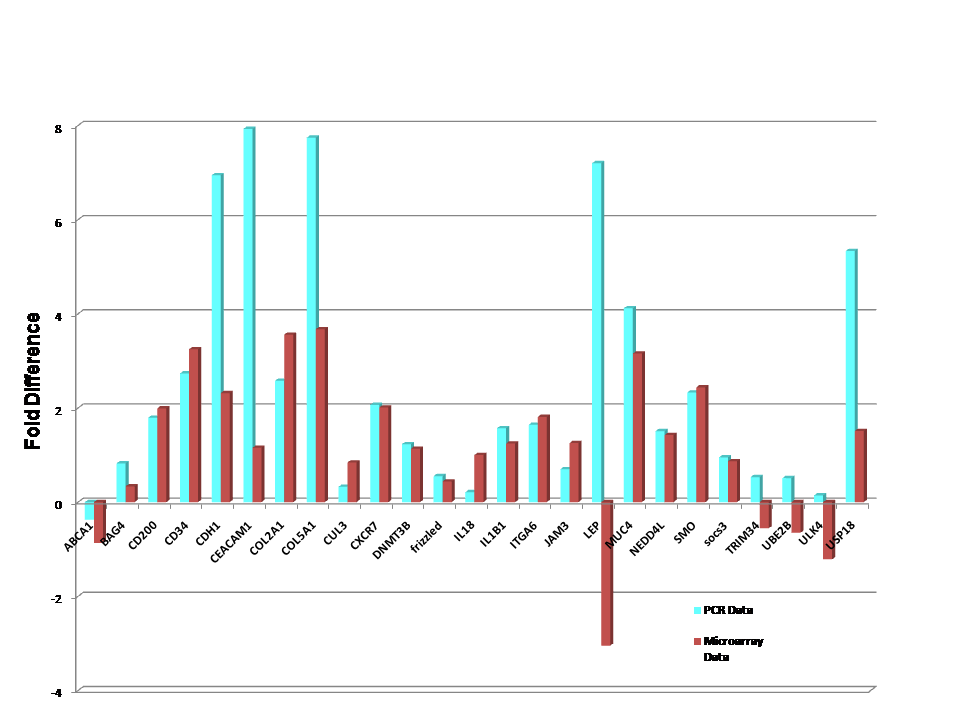


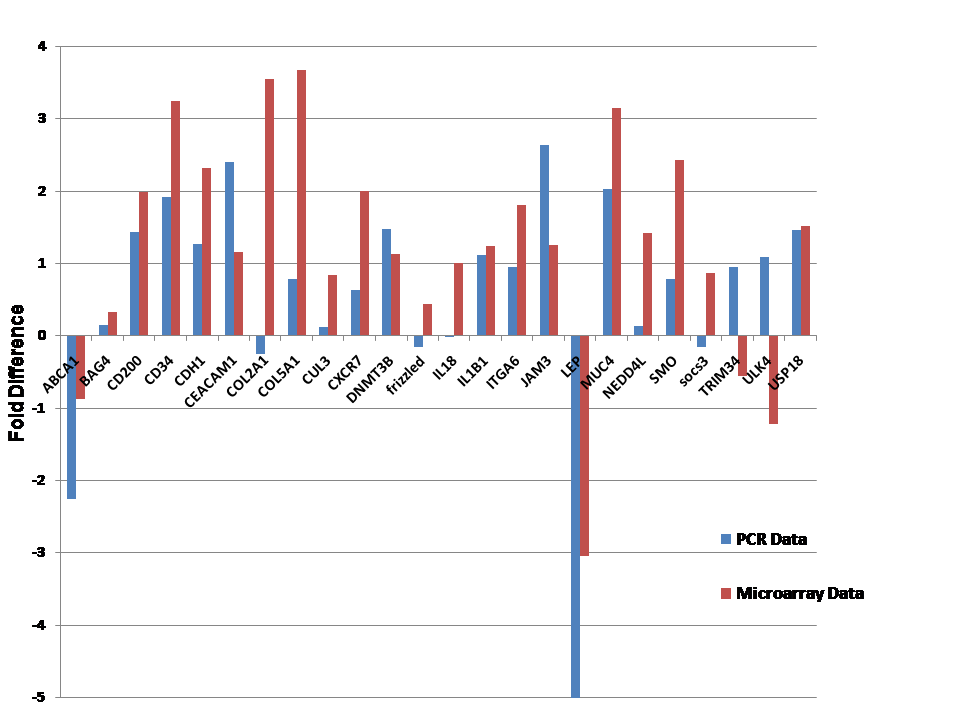
**Figure F**: **Validation of a set of microarray predicted gene expression in a second cohort of paired matched samples (n=10) that were available at diagnosis and at relapses.**  The bar graph shows the comparison between the relative fold difference in the selected 25 genes between the initial diagnosis and at the subsequent relapse of 10 patients. 18 of the 25 genes evaluated by RQ-PCR in these cases had the regulation altered in the same direction as predicted by the microarray data from the initial cohort.

**Results D: The role of microenvironment interaction mediated resistance against ATO *in vitro:***

**Figure G: Protective effect of stromal cell co-culture of NB4 cells against ATO.** The bar graph shows the mean viability percentage of the NB4 cells when cultured with or without stromal cells (MSCs) and at varying concentrations of ATO incubated for 48 hours prior to apoptosis assay. There is a significant protective effect in NB4 cells by MSC co-culture to ATO induced apoptosis (N=9).


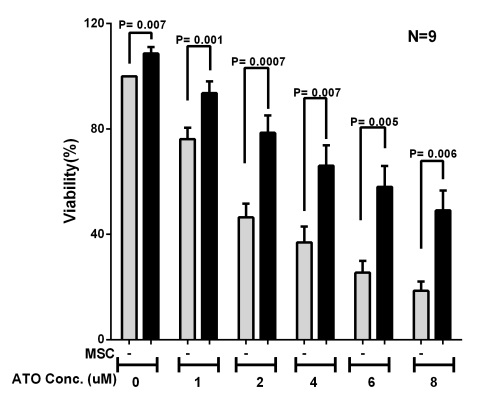


**Figure H: Effect of stromal cell interaction on cell cycle and cell proliferation assay on NB4 cells in the stromal cell co-culture system.** [A] Cell cycle analysis NB4 cells with and without co-culture with HS-5 cells for 48H stained with PI [B]. The NB4 cells were stained with CFSE and cultured with and without HS-5 cells, for 24, 48H and 72 H respectively. All the experiments were reported as an average of at least 3 independent experiments.


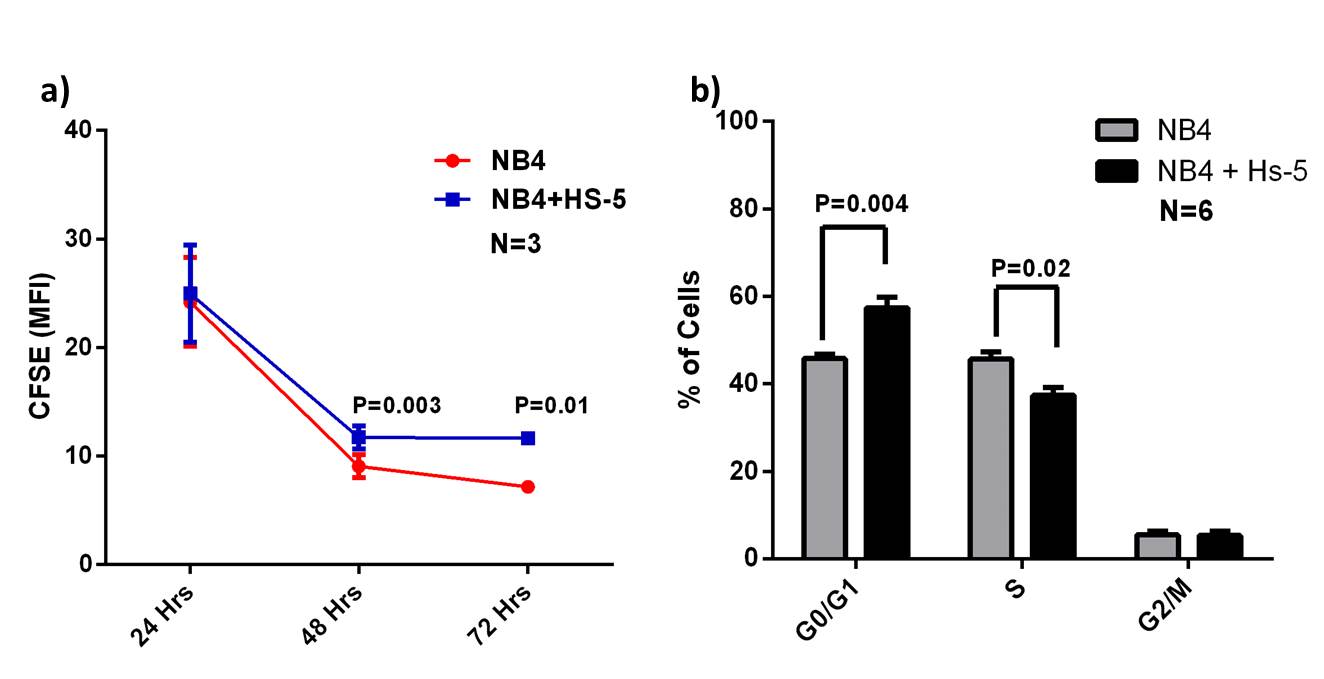


**SI File: Discussion:**

**Microarray analysis:**

Some of the pathways dysregulated in relapse are explored in detail to understand mechanistic pathways, as to how some malignant promyelocytes develop resistance.

**Adhesion pathway:**

The most prominently differentially regulated pathway between the diagnosis and relapse group was the adhesion pathway. Key genes significantly up regulated in relapse were VCAM1, ITGA6, ITGB4, ITGB6, JAM3, MUC4, Selectins, laminins, collagen type V,VI, VII and VIII, cadherins 1,2,4, 22 and 26 and claudins. Cell adhesion mediated resistance of malignant cells by modulating anti-apoptotic gene expression and favoring quiescence is known to result in drug resistance (4-6). The cytokines such as IL-8, FGF6, FGF17,FGFR3, IL-1β, IL-10, IL-12A, IL-17D,TGF-β1 and TGF-β3 are up regulated in relapse which are known play important roles in survival, and proliferation of malignant cells and potentially contribute to increased EM-DR (7-11). These observations suggest that EM-DR could potentially play a major role in subsequent relapse in APL following treatment with ATO.

**Pro-survival and anti-apoptotic pathways**

Nodal molecules of mitogen activated protein kinase (MAPK) such as small GTP binding proteins, Ras and Rap1(RAB15,2 8,38, RAP2,RASD1AND RASLFL12) and Rho GTPase genes like (FARP1, DNMBP, ARGGAP22, ADRALA) are up regulated in relapse which are known to activate PI3K-AKT pathway. p21-activated kinase effector gene Rac1/Cdc42 is dysregulated in relapse which is a key downstream effector of PI3K (12, 13). Transcription factors up regulated at the time of relapse were, c-MYC, FOS, HOXA1, HOXA9, HOXC9, FOX family genes and NF-kB. The NF-kB target genes like, VCAM1, NKIRAS1 were also up regulated in relapse.

***Hematopoietic stem and progenitor markers:***

The important effector molecule of Wnt pathway, the β-catenin regulatory components like glycogen synthase kinase (GSK3β) and casein kinase (CK1) are dysregulated in relapse patients. Other genes related to Wnt signalling (Cyclin D, c-MYC, EPHA3 and Frizzled7 ) as well as TGFβ (TGFβ receptors and SMAD4) were dysregulated in relapsed group. Two other signaling pathways that are hematopoietic stem cells markers which are dysregulated in relapse are hedgehog pathway and notch signaling(14). Notch gene is reported to mediate self renewal capacity in PML-RARA induced progenitor cells(15). The early HSC markers such as CD34 were increased and mature markers such as CD13, CD38 and CD44 were decreased significantly in the relapsed group in our microarray data and validated by our immunophenotypic observation.

In summary, mechanism of resistance and subsequent relapse in RAPL following treatment with ATO is probably multi-factorial. Based on our observations in this study we propose a model for ATO resistance (Figure S10). Prominent in this model is the possible expansion of a leukemia initiating compartment and increased EM-DR. A better understanding of ATO resistance would allow the rational design of clinical trials of ATO combined with other agents to improve the efficacy and overcome resistance.

**Figure I: Hypothetical model of ATO resistance based on our observations in this study**

Panel A represents the leukemia compartment in a case with newly diagnosed patients with APL. The proportion of cells in the leukemia initiating compartment and the relative EM-DR afforded is low enough to result in the cure of 80% of cases treated with single agent ATO. Panel B illustrates the situation in the relapsed cases where the proportion of cells in the leukemia initiating compartment (LIC) is variably increased as is the increase (again variable) in the EM-DR to ATO (It should be noted that additional experiments are required to validate concept of an expanded LIC in relapsed APL). The net result of these combined factors is an increased risk of relapse following treatment with ATO. In the absence of clonally acquired mutation leading to resistance to ATO (rarely reported), sensitivity to ATO is retained in the bulk population and repeated complete remissions can be achieved with this agent even after multiple relapses though cure becomes increasingly unlikely with this approach (especially so with single agent ATO).

**References:**

1. Mitelman F. ISCN 1995: An International System for Human Cytogenetic Nomenclature. Basel, Switzerland: Karger; 1995.

2. Chen GQ, Shi XG, Tang W, et al. Use of arsenic trioxide (As2O3) in the treatment of acute promyelocytic leukemia (APL): I. As2O3 exerts dose-dependent dual effects on APL cells. Blood 1997;89:3345-53.

3. Morelli E, Mascherpa MC, Scarano G. Biosynthesis of phytochelatins and arsenic accumulation in the marine microalga Phaeodactylum tricornutum in response to arsenate exposure. Biometals 2005;18:587-93.

4. Konopleva M, Konoplev S, Hu W, Zaritskey AY, Afanasiev BV, Andreeff M. Stromal cells prevent apoptosis of AML cells by up-regulation of anti-apoptotic proteins. Leukemia 2002;16:1713-24.

5. Lane SW, Scadden DT, Gilliland DG. The leukemic stem cell niche: current concepts and therapeutic opportunities. Blood 2009;114:1150-7.

6. Roozendaal R, Mebius RE. Stromal Cell-Immune Cell Interactions. Annu Rev Immunol 2010.

7. Zhukareva V, Obrocka M, Houle JD, Fischer I, Neuhuber B. Secretion profile of human bone marrow stromal cells: donor variability and response to inflammatory stimuli. Cytokine 2010;50:317-21.

8. Oh JY, Kim MK, Shin MS, Wee WR, Lee JH. Cytokine secretion by human mesenchymal stem cells cocultured with damaged corneal epithelial cells. Cytokine 2009;46:100-3.

9. Liu CH, Hwang SM. Cytokine interactions in mesenchymal stem cells from cord blood. Cytokine 2005;32:270-9.

10. Kilroy GE, Foster SJ, Wu X, et al. Cytokine profile of human adipose-derived stem cells: expression of angiogenic, hematopoietic, and pro-inflammatory factors. J Cell Physiol 2007;212:702-9.

11. Majumdar MK, Thiede MA, Haynesworth SE, Bruder SP, Gerson SL. Human marrow-derived mesenchymal stem cells (MSCs) express hematopoietic cytokines and support long-term hematopoiesis when differentiated toward stromal and osteogenic lineages. J Hematother Stem Cell Res 2000;9:841-8.

12. Yang W, Klaman LD, Chen B, et al. An Shp2/SFK/Ras/Erk signaling pathway controls trophoblast stem cell survival. Dev Cell 2006;10:317-27.

13. Sundberg-Smith LJ, Doherty JT, Mack CP, Taylor JM. Adhesion stimulates direct PAK1/ERK2 association and leads to ERK-dependent PAK1 Thr212 phosphorylation. J Biol Chem 2005;280:2055-64.

14. Beauchamp EM, Ringer L, Bulut G, et al. Arsenic trioxide inhibits human cancer cell growth and tumor development in mice by blocking Hedgehog/GLI pathway. J Clin Invest 2011;121:148-60.

15. Nicole R. Grieselhuber JMK, Angela M. Verdoni and Timothy J Ley. Activation of Notch Signaling Is An Early Event in the Development of PML-Rara-Induced Acute Promyelocytic Leukemia (APL). Annual Society of Hematology 2011;2468
